# Simulating the Impact of Zoning Policies on Equitably Reducing Neighborhood Tobacco Retailer Availability

**DOI:** 10.1007/s11524-026-01119-6

**Published:** 2026-07-21

**Authors:** Amanda Y. Kong, Meng Chen, Lily Herbert, Kurt M. Ribisl, Daniel P. Giovenco, Bryce C. Lowery, Mark Meaney, Elizabeth Chery-Mullen

**Affiliations:** 1 Department of Social Sciences and Health Policy, Division of Public Health Sciences, Wake Forest University School of Medicine, Winston-Salem, NC USA; 2 Department of Psychology, Dana and David Dornsife College of Letters, Arts and Sciences, The University of Southern California, Los Angeles, CA USA; 3 Department of Health Behavior, UNC Gillings School of Global Public Health, Chapel Hill, NC USA; 4 Department of Sociomedical Sciences, Columbia University Mailman School of Public Health, New York, NY USA; 5 School of Public Affairs, San Diego State University, San Diego, CA USA; 6 Public Health Law Center, St. Paul, MN USA; 7 Chronic Disease and Prevention Services, Oklahoma State Department of Health, Oklahoma City, OK USA

**Keywords:** Zoning, Tobacco retailer density, Point of sale, Health equity, Preemption

## Abstract

Living in a neighborhood with greater tobacco retailer availability (TRA) is associated with tobacco use, and there are persistent neighborhood racialized and socioeconomic inequities in TRA. Zoning could be leveraged as an innovative policy tool to equitably reduce TRA. In this study, we simulated the effect of zoning policies on reducing inequities in TRA in Oklahoma City (OKC) and Tulsa (Oklahoma). We spatially joined locations of tobacco retailers, zoning areas, and census tract sociodemographic characteristics (year 2023). We conducted 500 simulations to randomly remove 5–90% (in increments of 5%) of tobacco retailers from different zones and then calculated the average count of tobacco retailers left in each city across all simulations (i.e., post-policy count). We examined the policy effects by comparing the baseline (i.e., no removal of tobacco retailers) to the post-policy count by neighborhood sociodemographic quintile (Q1 = lowest; Q5 = highest). Policies that limited the number of tobacco retailers in commercial zones had pro-equity effects. For example, in OKC, with each additional percentage of tobacco retailers removed from commercial zones, one additional tobacco retailer was removed from Q5 as compared to Q1 percentage of Black residents (Q5 −1.02; 95% CI −1.63, −0.41). However, some zoning policies had little effect or widened inequities. We provide promising evidence that tailored zoning policies may reduce inequities in TRA though zoning policies may not eliminate inequities. Local jurisdictions, especially those in preempted states, can conduct equity assessments to inform the design of pro-equity zoning-based policies, which may complement, but not replace, other TRA reduction policies.

**Supplementary Information:**

The online version contains supplementary material available at https://doi.org/10.1007/s11524-026-01119-6.

## Introduction

Tobacco use remains the leading cause of preventable death[1, 2], and racialized and socioeconomic inequities in tobacco use and related diseases persist[3–5]. The tobacco retail environment has increasingly become an important point of intervention for decreasing tobacco use[6, 7] and specifically for ameliorating tobacco-related inequities[8, 9]. In places with higher tobacco retailer availability (TRA), there may be lower travel costs to obtain tobacco products and greater marketing exposure, cueing tobacco use behaviors[10–13]. Indeed, studies indicate that increased TRA is associated with both youth and adult tobacco use and health outcomes[14–18]. There are also persistent racialized and socioeconomic inequities in TRA whereby neighborhoods with a greater composition of racially and ethnically minoritized individuals and those with lower socioeconomic status experience greater TRA[19]. As such, policies to reduce neighborhood TRA are needed to reduce tobacco-related health inequities[8, 9, 20, 21].

To address inequities in TRA, several cities have implemented licensing-based policies, such as limiting the number of tobacco retailer licenses overall, as well as in particular neighborhoods or districts[22–25]. Licensing policies grant business owners who hold the license the privilege to sell tobacco products. However, state-level preemption prohibits many local governments from enacting a spectrum of tobacco control policies, and preemption is a significant barrier to tobacco control that may reify inequities[26–30]. As of June 2024, approximately 17 states preempt local governments from regulating some aspect of tobacco retail licensing[31], creating legal hurdles to implementing tobacco retailer reduction policies. For example, one promising local policy is to “cap” or limit the number of retailers that have a license to sell tobacco products[32, 33]. However, in places where retail licensing is preempted, local jurisdictions may not be able to implement this policy as it is centered on regulating the actual license to sell, a power reserved to the state. Additionally, some retailer reduction policies, such as pharmacy bans, may actually exacerbate inequities in TRA as pharmacies are more present in neighborhoods with higher socioeconomic status and a lower composition of racially and ethnically minoritized residents[20, 32, 34, 35]. Public health policies to reduce neighborhood TRA equitably are needed to prevent and reduce tobacco use and related disease, especially in preempted states.

Oklahoma has some of the most restrictive preemption policies in the nation, which include preserving exclusive authority over tobacco retailer licensing to the state[31], prohibiting localities from implementing any policies pertaining to the provision of tobacco licenses. However, Oklahoma does not preempt “any agency or political subdivision from exercising its lawful authority to regulate zoning or land use”[36]. Zoning regulates how land can be used and developed and plays a significant role in shaping built environments[37, 38]. While zoning regulates the size, shape, and placement of structures on a parcel of land, a primary function of zoning is to regulate land use whereby parcels of land are designated for certain uses and operations (e.g., residential, industrial)[39]. Zones are often patterned by neighborhood sociodemographic characteristics due to racist and discriminatory practices[38, 40, 41]. For example, antidensity zoning policies that primarily limit home growth and development, especially in terms of housing options, are more common in affluent and White neighborhoods and can reduce affordable housing, producing and strengthening both racialized and socioeconomic residential segregation [40, 41]. As TRA is also inequitably distributed by neighborhood sociodemographic characteristics (i.e., greater TRA in lower socioeconomic neighborhoods and those with a higher proportion of Black and Hispanic or Latine residents)[19], zoning could be leveraged as an innovative policy tool to more equitably regulate the places and land where retailers are permitted to sell tobacco products.

Since the first study assessing neighborhood inequities in TRA[42], numerous studies have documented inequities and concluded that zoning and other land use tools could be used to reduce TRA[43–52]. However, few studies have explicitly examined relationships between land use and TRA. One study evaluating the impact of San Francisco’s (California) licensing-based tobacco retailer reduction policy found that zones were inequitably distributed across districts and may contribute to inequitable policy impacts[53]. The authors recommended that zones be assessed prior to the implementation of policies[53]. Another study in Wilmington, Delaware (described as having “high poverty rates and a majority Black population”), found more tobacco retailers in medium and high-density residential zones and speculated, but did not empirically document, that reducing tobacco retailers in these zones may reduce retailer exposure[54]. Finally, our previous work in Oklahoma City (OKC, Oklahoma [OK]) and Tulsa (OK) found greater TRA in commercial zones near residential areas for census tracts with greater socioeconomic disadvantage, a higher percentage of Black and Hispanic/Latine residents, and a lower percentage of White residents[55].

Regulating the zones where retailers are permitted or prohibited from selling tobacco may be a policy tool that can be intentionally designed to reduce neighborhood inequities in TRA, especially in those states where local tobacco retail licensing is preempted[33, 56]. While a retailer may be required to have a permit to sell (issued by the state), local zoning ordinances may regulate where the retailer can operate with their license to sell tobacco. The overall objective of this study was to simulate policies that cap or limit the number of tobacco retailers within certain zones and compare changes in observed neighborhood inequities in TRA pre- vs. post-policy in OKC and Tulsa.

## Methods

A full description of data sources is described elsewhere[55], but we provide a brief overview below.

### Study Geographies and Unit of Analysis

We conceptualize neighborhoods as census tracts (which typically range from 1200 to 8000 residents), and the analytic sample included all 268 and 151 census tracts in OKC and Tulsa, respectively (year 2023). Census tract represents the unit of analysis for the models described below.

### Neighborhood Sociodemographic Characteristics

We downloaded census tract-level sociodemographic characteristics from the 2019–2023 American Community Survey[57], including the percentage of non-Hispanic Black or African American residents (% Black), percentage Hispanic or Latino residents (% Hispanic/Latine), percentage non-Hispanic White residents (% White), percentage of residents living below the federal poverty line (FPL), median household income, and the Gini index, which ranges from 0 (i.e., all households have equal income) to 1 (i.e., a single household has all income). We prioritized these sociodemographic variables as they are most commonly used in studies examining neighborhood inequities in tobacco retailer availability[19]. We quintiled each sociodemographic variable where Q1 and Q5 represent the lowest and highest value of a sociodemographic variable, respectively (e.g., Q1 of % Black and median household income represents neighborhoods with the lowest percentage of % Black residents and lowest median household income). We chose to utilize quintiles as previous literature indicates nonlinear neighborhood inequities in tobacco retailer availability[19].

### Zoning Designations

We downloaded publicly available zoning data in July–August 2023 for OKC (*n* = 21,652 zoning parcels) and Tulsa (*n* = 4103 zoning parcels). Our analysis focused on zoning designations that had at least 10 tobacco retailers in at least one quintile of one of the above neighborhood sociodemographic characteristics (i.e., either Q1, Q2, Q3, Q4, or Q5 of any sociodemographic variable had at least 10 tobacco retailers) to identify zoning designations that would be most impacted by zoning-based tobacco control policies (i.e., targeted policy zones). For the present analysis, we included five targeted policy zones in OKC: C-3 (Community Commercial), C-4 (General Commercial), I-1 (Light Industrial), I-2 (Moderate Industrial), and PUD (Planned Unit Development). Additionally, we included two in Tulsa: CH (Commercial High Intensity) and CS (Commercial Shopping). Approximately 87% and 79% of tobacco retailers fell within these specific zoning designations in OKC and Tulsa, respectively. A full description of these zones is described elsewhere: https://ars.els-cdn.com/content/image/1-s2.0-S1353829225000863-mmc1.pdf[55]. In our previous work, we documented city-wide racialized and socioeconomic inequities in TRA in OKC and Tulsa, demonstrating the importance of pro-equity tobacco retailer reduction policies in these two places[55].

### Tobacco Retailers

We obtained a list of licensed OK tobacco retailers and their respective addresses (February 2023) from the OK State Department of Health. After geocoding and cleaning the list (e.g., Google-verifying addresses with a poor geocoding match score), we included 577 and 321 licensed tobacco retailers in OKC and Tulsa, respectively. We spatially intersected each tobacco retailer location with (a) zoning parcels and (b) census tract sociodemographic variables, which resulted in a retailer being assigned a single zoning parcel designation and its respective census tract sociodemographic variable quintile.

### Analysis

All analyses were conducted in R. We conducted 500 simulations to randomly remove 5–90% (in increments of 5%) of tobacco retailers from each of the targeted policy zones, described above, for each city. While reducing the number of tobacco retailers by a large percentage (e.g., 90%) may be politically infeasible, there are jurisdictions that have implemented complete retail sales bans on tobacco products (e.g., Beverly Hills[58] and Manhattan Beach, California[59]), and a gradual phasing out of retail tobacco sales has been of interest in other places, such as Australia[60]. Additionally, some US jurisdictions that have implemented tobacco retailer reduction policies have purposefully included language to make these policies more stringent in the future. For example, New York City initially capped its number of tobacco retailers to 50% of the total number of tobacco licenses, but the policy includes language allowing the city to evaluate this 50% metric every 2 years, allowing the city to further reduce tobacco licenses[61]. For these reasons, we extend our simulations to 90%.

We determined the number of simulation replications by increasing runs until the estimated mean count of remaining tobacco retailers showed negligible change with additional iterations, and the Monte Carlo standard error (MCSE) fell below a threshold (MCSE < 0.08). Both criteria were met at 500 replications. After simulating the random removal of tobacco retailers from each targeted policy zone, we then examined the impact of simulated policies across each city, comparing the baseline or pre-policy (i.e., no random removal of tobacco retailers) to the post-policy count of tobacco retailers by census tract sociodemographic quintiles in OKC and Tulsa. In essence, this allows us to simulate what a potential zoning-based “cap” tobacco control policy would look like in each city. For example, OKC had 302 tobacco retailers in C-3 zones[55]. A 5% reduction in tobacco retailers in C-3 zones would result in a reduction of 15 tobacco retailers, equivalent to a zoning-based policy that caps or limits the number of tobacco retailers in C-3 zones to 287 tobacco retailers. Similarly, a 10% and 15% reduction would result in 30 and 45 fewer tobacco retailers or a policy cap that limits tobacco retailers in C-3 zones to 272 and 257, respectively.

In this study, we define an “inequity in TRA” as neighborhoods with lower socioeconomic status/greater income inequality or a higher proportion of Black/Latine residents having greater neighborhood TRA. To examine the equity impact of our simulated policies, we fit linear regression models to examine if the tobacco retailer count decreased more and at a quicker rate in census tracts experiencing inequities in TRA (i.e., a pro-equity policy). To do this, we fit models that included an interaction term between each sociodemographic quintile (where Q1 represents the reference group) and the percentage removal of tobacco retailers. These interaction terms represent the linear slope for each quintile as compared to Q1 where a negative number indicates a greater decline in tobacco retailer count for the respective quintile as compared to Q1, but a positive number represents a less steep decline for the respective quintile as compared to Q1 (see model equation below). Said another way, the Q2–Q5 interaction effects ($\gamma^{\prime }s$ in the model equation below) test whether the rate of decline in the number of remaining tobacco retailers post-policy (from 5 to 90% removal) differs from the rate in Q1. We additionally report the regression coefficient for Q1 ($${\beta}_{1}$$), which tests whether there is a statistically significant change in the number of remaining tobacco retailers post-policy (from 5 to 90% removal) compared to the baseline number of tobacco retailers (pre-policy, $${\beta}_{0}$$).

$$T{RA}_i=\beta_0+\beta_1R_i+\sum\nolimits_{q=2}^5\beta_qQ_{qi}+\sum\nolimits_{q=2}^5\gamma_q\left(R_i\times Q_{qi}\right)+\varepsilon_i$$

In the equation above, $T{RA}_{i}$ is the number of remaining tobacco retailer for observation *i* in the simulations. ${R}_{i}$ is the proportion of removal associated with this observation, and ${Q}_{qi}$ is an indicator variable showing whether observation i belongs to group ${Q}_{q}$ (e.g., ${Q}_{2i}=1$ if observation i is in Q2, and ${Q}_{2i}=0$ otherwise). ${\varepsilon}_{i}$ is the residual associated with observation *i.*

Finally, we calculated and plotted the model-implied total tobacco retailer count (with 95% confidence intervals) remaining in each city across quintiles of each sociodemographic variable post-policy to visualize potential policy impacts.

## Results

### Oklahoma City (OKC)

**Fig. 1:**
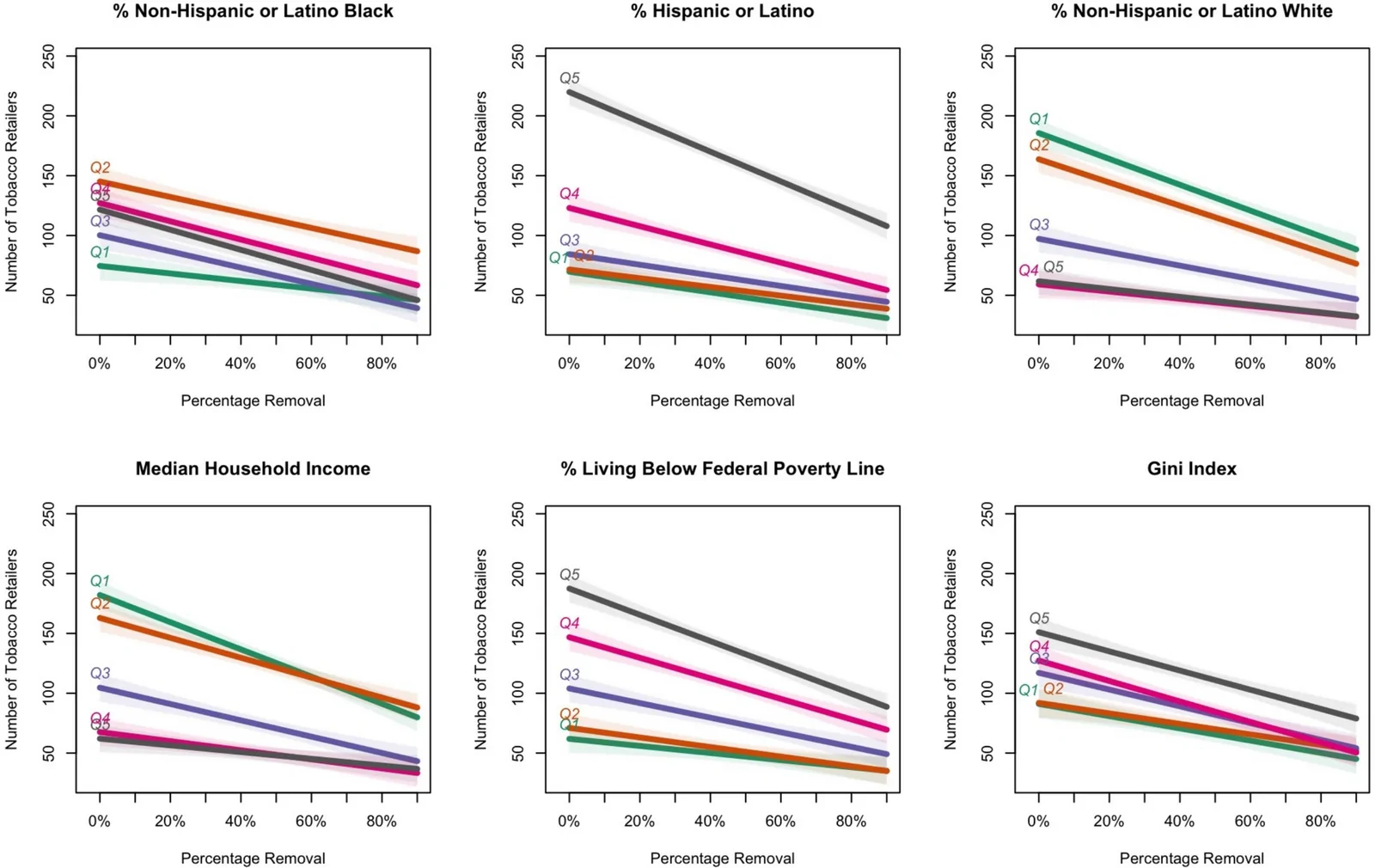
Combined Community Commercial (C-3) and General Commercial (C-4) tobacco control policy cap simulation: estimated count of tobacco retailers remaining pre- vs. post-policy simulation by sociodemographic variable quintiles, Oklahoma City, Oklahoma. Note: Quintile (Q)1 represents the lowest value of the sociodemographic variable while Q5 represents the highest value of the sociodemographic variable

In OKC, policies that limit the number of tobacco retailers in commercial zones (C-3, C-4) would have a pro-equity effect, and this effect was slightly more pronounced for policies that limited TRA in both community commercial (C-3) and general commercial (C-4) zones (rather than just either zoning type). For example, Q5 of percentage of Black residents was associated with a −1.00 (95% CI −1.63, −0.37) reduction in the linear slope of remaining estimated tobacco retailers in C-3 zones (as compared to Q1 percentage of Black residents, Table 1). This same pattern of a steeper decline in TRA for census tracts with a greater percentage of Black residents was consistent across all quintiles for policies that limited the number of retailers in C-3 and C-4. Figure 1 visualizes these policy effects (i.e., estimated effect sizes or slopes). For example, the largest inequity pre-policy was between Q2% Black (148 tobacco retailers) and Q1 (pre-policy count: 76 retailers). Our models estimated that a policy reduction of 20% of tobacco retailers in C-3 and C-4 combined would result in a post-policy count of 70 tobacco retailers in Q1 (absolute difference: 6 retailers; percentage change: 7.89% decrease) and 135 tobacco retailers in Q2 (absolute difference: 13 tobacco retailers; percentage change: 8.78%), indicating a steeper decline for Q2 as compared to Q1 (where Q2 *B* = −0.62; 95% CI −1.23, −0.02).

**Table 1:** Simulation modeling results testing whether the rate of decline in the number of remaining tobacco retailers post-policy (from 5 to 90% removal) differs from the rate of change in quintile (Q)1

Targeted policy zone (# of tobacco retailers in zone) and sociodemographic quintile	Census tract-level sociodemographic variable					
	% Black residents	% Hispanic/Latine residents	% White residents	Median household income	% living below FPL	Gini index
	*B* (95% CI)	*B* (95% CI)	*B* (95% CI)	*B* (95% CI)	*B* (95% CI)	*B* (95% CI)
Oklahoma City (OKC)						
Community Commercial, C3 (*n* = 302)						
1 (ref)	**−0.59 (−1.03, −0.15)**	**−0.80 (−1.21, −0.39)**	**−1.81 (−2.23, −1.39)**	**−2.01 (−2.43, −1.59)**	**−0.54 (−0.96, −0.12)**	**−0.91 (−1.35, −0.46)**
2	−0.39 (−1.01, 0.24)	0.19 (−0.38, 0.77)	0.13 (−0.46, 0.72)	**0.72 (0.12, 1.32)**	−0.21 (−0.81, 0.39)	0.21 (−0.42, 0.84)
3	−0.58 (−1.20, 0.05)	−0.02 (−0.59, 0.56)	**0.81 (0.22, 1.40)**	**0.78 (0.18, 1.38)**	−0.48 (−1.07, 0.12)	−0.34 (−0.97, 0.29)
4	**−0.71 (−1.34, −0.09)**	−0.54 (−1.12, 0.04)	**1.26 (0.66, 1.85)**	**1.29 (0.69, 1.89)**	**−0.90 (−1.50, −0.30)**	**−0.65 (−1.28, −0.02)**
5	**−1.00 (−1.63, −0.37)**	**−1.28 (−1.86, −0.71)**	**1.25 (0.65, 1.84)**	**1.51 (0.91, 2.11)**	**−1.39 (−1.99, −0.79)**	−0.42 (−1.05, 0.21)
Community and General Commercial, C-3 and C-4 combined (*n* = 330)						
1 (ref)	**−0.60 (−1.03, −0.17)**	**−0.81 (−1.21, −0.41)**	**−2.04 (−2.45, −1.63)**	**−2.14 (−2.56, −1.73)**	**−0.56 (−0.97, −0.15)**	**−0.96 (−1.40, −0.53)**
2	**−0.62 (−1.23, −0.02)**	0.12 (−0.44, 0.69)	0.21 (−0.37, 0.78)	0.57 (−0.01, 1.16)	−0.20 (−0.78, 0.39)	0.13 (−0.48, 0.75)
3	**−0.68 (−1.29, −0.07)**	−0.02 (−0.59, 0.54)	**0.98 (0.41, 1.56)**	**0.86 (0.27, 1.44)**	**−0.59 (−1.17, −0.01)**	−0.36 (−0.97, 0.26)
4	**−0.84 (−1.45, −0.23)**	**−0.63 (−1.19, −0.06)**	**1.47 (0.89, 2.05)**	**1.43 (0.84, 2.01)**	**−1.06 (−1.64, −0.48)**	**−0.65 (−1.27, −0.03)**
5	**−1.02 (−1.63, −0.41)**	**−1.58 (−2.15, −1.02)**	**1.41 (0.83, 1.99)**	**1.60 (1.02, 2.19)**	**−1.55 (−2.14, −0.97)**	−0.58 (−1.19, 0.04)
Light Industrial, I-1 (*n* = 78)						
1 (ref)	−0.26 (−0.77, 0.25)	−0.02 (−0.49, 0.46)	**−0.56 (−1.04, −0.07)**	−0.27 (−0.76, 0.22)	−0.06 (−0.54, 0.43)	−0.19 (−0.71, 0.33)
2	−0.08 (−0.80, 0.65)	0.00 (−0.67, 0.67)	0.37 (−0.31, 1.06)	−0.17 (−0.86, 0.52)	0.06 (−0.64, 0.75)	−0.11 (−0.84, 0.62)
3	0.20 (−0.52, 0.93)	0.00 (−0.67, 0.67)	0.52 (−0.16, 1.21)	0.19 (−0.51, 0.88)	0.04 (−0.66, 0.73)	0.13 (−0.60, 0.86)
4	0.16 (−0.57, 0.89)	−0.06 (−0.73, 0.62)	0.56 (−0.13, 1.25)	0.23 (−0.47, 0.92)	−0.30 (−1.00, 0.39)	0.07 (−0.66, 0.81)
5	0.20 (−0.53, 0.93)	−0.66 (−1.34, 0.01)	0.54 (−0.15, 1.23)	0.27 (−0.43, 0.96)	−0.32 (−1.02, 0.37)	0.04 (−0.70, 0.77)
Moderate Industrial, I-2 (*n* = 88)						
1 (ref)	−0.06 (−0.55, 0.44)	−0.06 (−0.51, 0.40)	−0.43 (−0.89, 0.04)	**−0.51 (−0.98, −0.04)**	−0.04 (−0.51, 0.43)	−0.27 (−0.76, 0.23)
2	−0.37 (−1.08, 0.33)	0.00 (−0.64, 0.64)	−0.07 (−0.73, 0.58)	0.08 (−0.59, 0.74)	−0.13 (−0.80, 0.53)	0.21 (−0.50, 0.92)
3	−0.19 (−0.89, 0.52)	−0.13 (−0.77, 0.51)	0.22 (−0.43, 0.88)	0.45 (−0.21, 1.12)	−0.17 (−0.83, 0.49)	0.02 (−0.69, 0.72)
4	−0.23 (−0.93, 0.48)	−0.27 (−0.91, 0.38)	0.43 (−0.23, 1.09)	0.45 (−0.21, 1.12)	−0.21 (−0.87, 0.46)	0.09 (−0.62, 0.80)
5	−0.09 (−0.80, 0.61)	−0.50 (−1.14, 0.15)	0.41 (−0.25, 1.07)	0.40 (−0.27, 1.06)	−0.47 (−1.14, 0.19)	−0.18 (−0.89, 0.53)
Planned Unit Development, PUD (*n* = 209)						
1 (ref)	−0.26 (−0.78, 0.26)	−0.22 (−0.69, 0.24)	−0.08 (−0.55, 0.40)	−0.13 (−0.62, 0.35)	−0.37 (−0.85, 0.11)	−0.17 (−0.69, 0.35)
2	−0.13 (−0.86, 0.61)	−0.13 (−0.79, 0.53)	−0.17 (−0.84, 0.51)	−0.06 (−0.74, 0.63)	0.04 (−0.65, 0.72)	−0.15 (−0.88, 0.59)
3	0.13 (−0.60, 0.87)	−0.09 (−0.75, 0.56)	−0.29 (−0.97, 0.38)	−0.14 (−0.82, 0.55)	0.03 (−0.65, 0.72)	−0.05 (−0.79, 0.68)
4	0.09 (−0.64, 0.83)	−0.04 (−0.70, 0.62)	−0.17 (−0.85, 0.51)	−0.20 (−0.88, 0.49)	0.24 (−0.44, 0.92)	−0.10 (−0.84, 0.64)
5	−0.04 (−0.77, 0.70)	0.13 (−0.53, 0.79)	−0.25 (−0.92, 0.43)	−0.23 (−0.92, 0.45)	0.30 (−0.39, 0.98)	−0.12 (−0.86, 0.62)
Tulsa						
Commercial High Intensity, CH (*n* = 85)						
1 (ref)	−0.29 (−0.78, 0.19)	−0.36 (−0.84, 0.13)	−0.39 (−0.88, 0.10)	**−0.57 (−1.05, −0.10)**	−0.06 (−0.55, 0.42)	−0.46 (−0.95, 0.04)
2	−0.21 (−0.90, 0.48)	0.08 (−0.61, 0.78)	0.15 (−0.54, 0.85)	0.20 (−0.47, 0.87)	−0.37 (−1.06, 0.32)	0.25 (−0.46, 0.96)
3	−0.28 (−0.97, 0.41)	−0.02 (−0.71, 0.67)	−0.14 (−0.84, 0.55)	−0.10 (−0.78, 0.57)	−0.48 (−1.17, 0.22)	0.12 (−0.59, 0.83)
4	−0.04 (−0.74, 0.65)	−0.32 (−1.01, 0.38)	−0.08 (−0.78, 0.61)	0.20 (−0.47, 0.87)	−0.41 (−1.10, 0.28)	−0.24 (−0.96, 0.47)
5	−0.04 (−0.73, 0.65)	−0.02 (−0.71, 0.68)	−0.01 (−0.71, 0.68)	0.51 (−0.17, 1.18)	−0.47 (−1.17, 0.22)	0.12 (−0.59, 0.83)
Commercial Shopping, CS (*n* = 141)						
1 (ref)	**−0.68 (−1.10, −0.26)**	**−0.55 (−0.97, −0.12)**	**−1.75 (−2.18, −1.32)**	**−1.70 (−2.11, −1.29)**	**−1.03 (−1.45, −0.61)**	**−0.93 (−1.36, −0.51)**
2	−0.41 (−1.02, 0.19)	−0.45 (−1.05, 0.16)	−0.18 (−0.79, 0.43)	−0.30 (−0.88, 0.29)	0.40 (−0.20, 1.00)	**−1.10 (−1.71, −0.49)**
3	−0.45 (−1.05, 0.15)	**−0.92 (−1.53, −0.31)**	**0.82 (0.20, 1.43)**	0.57 (−0.01, 1.15)	−0.20 (−0.80, 0.40)	0.00 (−0.61, 0.61)
4	**−1.08 (−1.69, −0.48)**	**−0.75 (−1.35, −0.14)**	**0.72 (0.11, 1.33)**	**0.87 (0.29, 1.46)**	−0.50 (−1.10, 0.10)	−0.23 (−0.84, 0.37)
5	**−1.00 (−1.60, −0.39)**	**−1.49 (−2.09, −0.88)**	**1.08 (0.47, 1.69)**	**1.00 (0.42, 1.59)**	**−0.87 (−1.47, −0.28)**	−0.33 (−0.94, 0.27)

Note: A negative estimate indicates a greater rate of decline in the number of remaining tobacco retailers post-policy for the respective quintile (Q2–Q5) as compared to Q1. Q1 estimates indicate whether there is a statistically significant change in the number of remaining tobacco retailers post-policy (from 5 to 90% removal) compared to the baseline number of tobacco retailers (pre-policy). Quintile (Q)1 represents the lowest value of the sociodemographic variable while Q5 represents the highest value of the sociodemographic variable. Bolding indicates statistical significance at alpha=0.05 (two-tailed)

Similarly, policies limiting the number of tobacco retailers in C-3 and C-4 zones were estimated to have a greater reduction for census tracts with the highest percentages of Hispanic/Latine residents (Table 1: Q4 −0.63, 95% CI, −1.19, −0.06; Q5 −1.58, 95% CI, −2.15, −1.02). While Q5 for percentage of Hispanic/Latine residents had the largest decline in TRA, these inequities are still largely present even after 90% removal of tobacco retailers (Fig. 1). Pro-equity effects were also observed for commercial-focused policies by percentage of White residents and median household income (but only for census tracts with the lowest percentage of White residents or median household income, Table 1). Though the inequity is never eliminated (lines cross), tracts with the greatest percentage of people living below the FPL experienced a much steeper decline in TRA than tracts with the lowest (Q5 −1.55, 95% CI −2.14, −0.97). We also estimated that zoning policies limiting the number of tobacco retailers in moderate industrial (I-2) zones significantly decreased TRA more for census tracts with the lowest median household income (Table 1).

Finally, while policies aimed at reducing TRA in planned unit development (PUD) zones did not result in statistically significant effect sizes, these estimates indicated that these types of policies may widen inequities for some neighborhoods (Table 1) as is also visualized by slopes close to 0 (flat lines) or steeper slopes for more privileged quintiles (Supplemental Figure 1). For example, quintiles with a greater percentage of residents living below the FPL had less of a decline in TRA as compared to Q1 (lowest percentage of FPL), represented through positive slope estimates for Q2–Q5 (Table 1). Visualizations for the other the other zones (i.e., I-1, I-2) can be found in Supplemental Figures 2 and 3.

### Tulsa

**Fig. 2:**
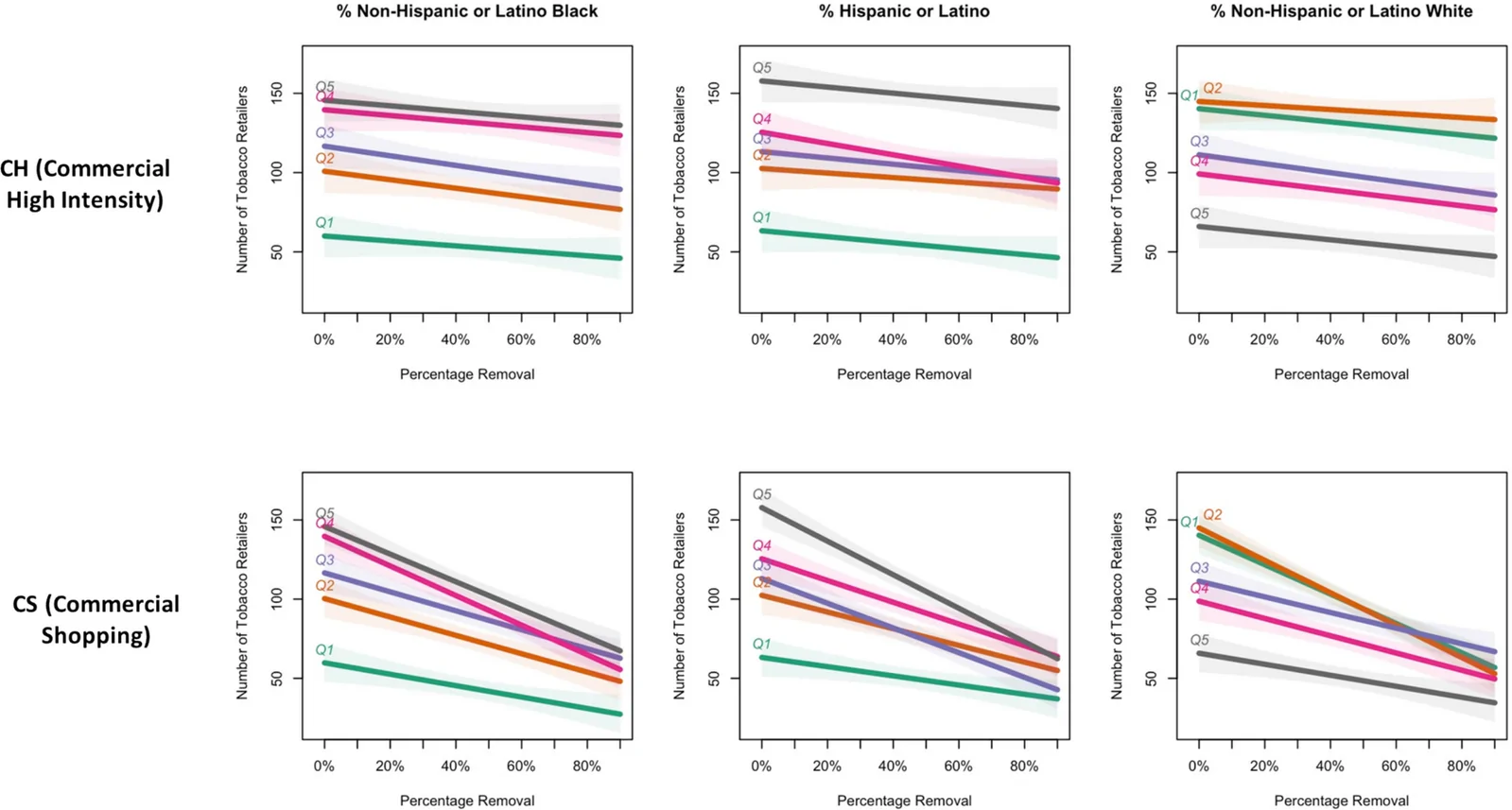
CH (Commercial High Intensity) and CS (Commercial Shopping) tobacco control policy cap simulation: estimated count of tobacco retailers remaining pre- vs. post-policy simulation by racial/ethnic variable quintiles, Tulsa, Oklahoma. Note: Quintile (Q)1 represents the lowest value of the sociodemographic variable while Q5 represents the highest value of the sociodemographic variable

**Fig. 3:**
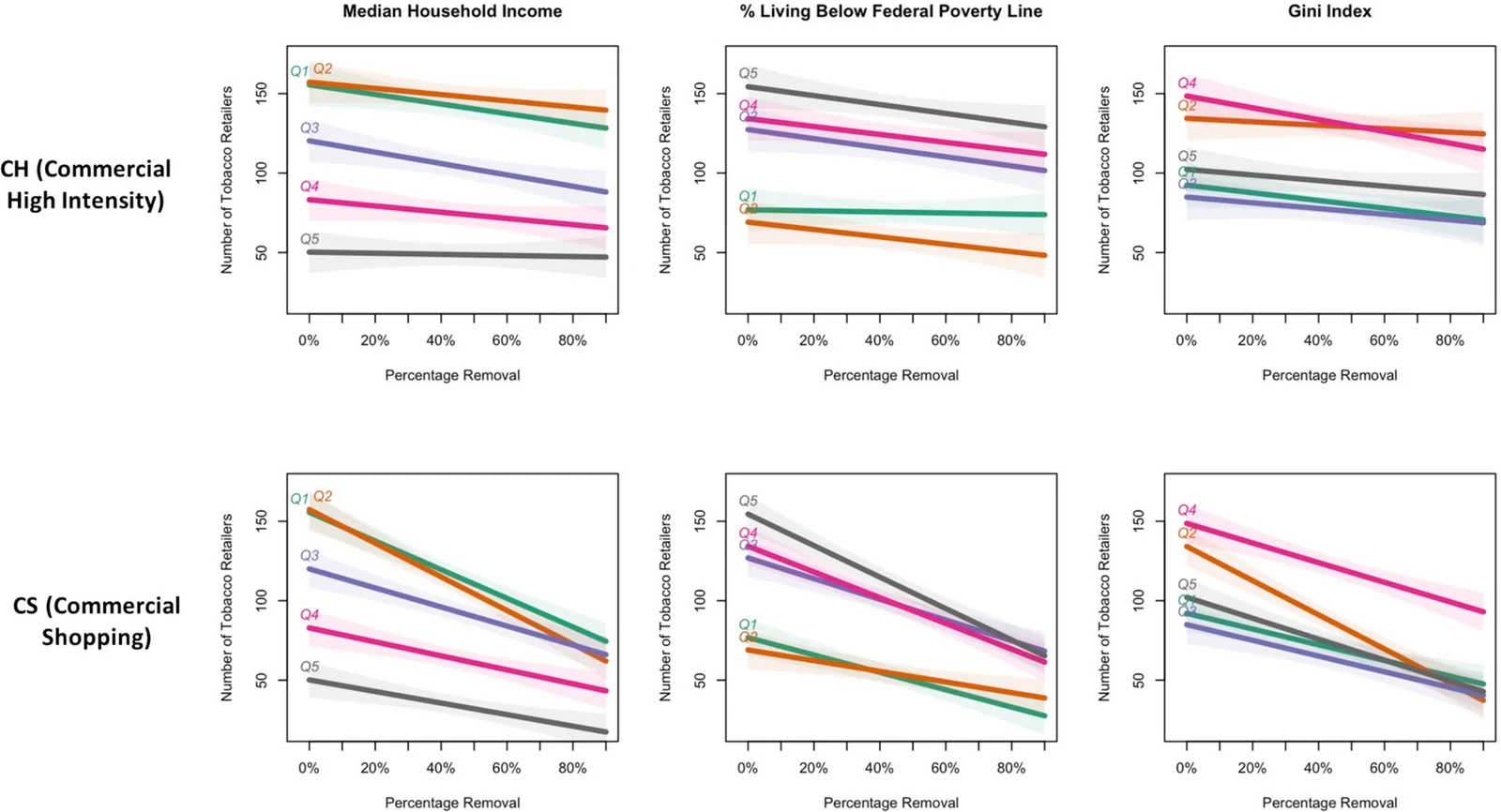
CH (Commercial High Intensity) and CS (Commercial Shopping) tobacco control policy cap simulation: estimated count of tobacco retailers remaining pre- vs. post-policy simulation by socioeconomic variable quintiles, Tulsa, Oklahoma. Note: Quintile (Q)1 represents the lowest value of the sociodemographic variable while Q5 represents the highest value of the sociodemographic variable

In Tulsa, estimates were generally negative (though not statistically significant) for commercial high intensity (CH) zones (Table 1, Figs. 2 and 3). In contrast, policies capping the number of tobacco retailers in commercial shopping (CS) zones would significantly reduce inequities for some quintiles of percentage of Black and Hispanic/Latine residents (Table 1, Figs. 2 and 3). For example, Q5 of percentage of Hispanic/Latine residents was associated with a −1.49 (95% CI −2.09, −0.88) steeper rate of decline in tobacco retailers (as compared to Q1 rate of decline, Table 1). We also observed that these policies would have pro-equity effects by median household income and for tracts with the highest (vs. lowest) percentage of residents living below the FPL (Q5 −0.87, 95% CI −1.47, −0.28).

## Discussion

Our study indicates that zoning policies that cap the number of retailers selling tobacco in certain zones have the potential to be pro-equity, that is, reduce overall TRA while also reducing or eliminating inequities in TRA. In our previous work, we found that certain types of commercial zones in OKC and Tulsa house a higher number of tobacco retailers, and this greater concentration of tobacco retailers is also associated with census tract sociodemographic characteristics, such as a greater percentage of Black and Hispanic/Latine residents and individuals with lower socioeconomic resources[55]. Additionally, zoning designations were patterned by census tract sociodemographic characteristics (e.g., C-3 zones were most prevalent in Q5% Black)[55], strengthening our hypothesis that zoning could be a mechanism for producing pro-equity declines in TRA. Consistent with the present study findings, we found that policies that targeted these types of zones led to a reduction or elimination in observed inequities. For example, in OKC, zoning policies that cap the number of tobacco retailers in C-3 and C-4 zones may have the greatest potential for reducing both racialized and socioeconomic inequities. Similarly, in Tulsa, policies that focus on CS (vs. CH) zones may have a more widespread impact on reducing inequities. In contrast [55], the zones that had little correlation with sociodemographic characteristics and TRA or indicated no inequity based on our previous work[55] resulted in simulated zoning policies that either had little effect on reducing TRA or widened inequities. For example, PUD-focused zoning policies in OKC may have little impact on reducing inequities, demonstrating the importance of assessing equity implications of policies before designing and implementing said policies. Our results are consistent with the few other studies examining zoning and land use patterns with tobacco retailer locations, such as those in California[53] and Delaware[54]. Our study may be among the first to explicitly examine these patterns and simulate the potential impact of zoning policies as a pro-equity policy tool.

Reductions in inequities were not always consistent across or within sociodemographic characteristics, consistent with studies examining neighborhood inequities in tobacco retailer availability[19]. This variability may be due to differences in land use and development and sociodemographic patterning. For example, PUD zones in OKC are a relatively new type of zone that allows more specialized and mixed land use and therefore may be more distributed throughout the city[55]. Similarly, in Tulsa, the strength and significance of inequities were more present in commercial shopping (CS) vs. commercial high intensity (CH) zones. This may be due to CS zones being more focused on “retail and personal services uses” and development, which better reflects tobacco retailer store types (e.g., convenience stores) whereas CH zones are focused more on using existing properties in the “core area of the city” while minimizing adverse residential impact[55]. Finally, while we use quintiles for our models, these are not inherently conceptually meaningful and local definitions of “high” vs. “low” population composition (for example) may be more informative and produce different results. Overall, given how localized zoning and land use policies are, tailoring of zoning policies and local coalition building and partnerships are needed to maximize potential pro-equity effects in select jurisdictions.

While we observed pro-equity effects for some simulated zoning policies where there were steeper declines in the estimated number of tobacco retailers in less privileged census tracts, we estimated that a large and potentially unfeasible percentage of retailers would have to stop selling tobacco to observe a meaningful decline or actual elimination of an inequity (i.e., where the estimated count of tobacco retailers in a neighborhood is now equal to or less than those in a more privileged neighborhood) across several sociodemographic characteristics and quintiles. In sensitivity tests (not shown), we limited our simulation to 75% removal and found trends to be similar, but some estimates as compared to Q1 (*n* = 11) were no longer statistically significant. Taken together, these findings demonstrate that while zoning policies can *reduce* inequities, zoning policies alone may have limited impact on *eliminating* inequities in TRA. This limitation reflects the stark existing inequities that are so large to begin with that even with a large percentage of retailers stopping the sales of tobacco in targeted zones, sociodemographic inequities across both OKC and Tulsa remain. Still, our study results indicate that zoning policies could be an important and effective pro-equity tobacco control strategy.

State-level preemption of tobacco control policies hinders the ability of localities to design and implement stronger tobacco control policies tailored to protect their communities and promote health. These often tobacco-industry backed preemptive policies stifle local tobacco control policies and likely contribute to tobacco use, tobacco-related disease, and impede health equity[30, 62–64]. For example, states with tobacco-related preemption have higher rates of smoking-attributable cancer mortality[64]. Repealing preemption on tobacco retailer licensing (as well as other tobacco control policies) in OK and other preempted states could allow localities to implement other policies beyond zoning, including tobacco retailer licensing-based policies, to more immediately reduce TRA (e.g., capping the number of tobacco licenses, prohibiting tobacco retailer licenses for retailers within 1000 feet of a school, and requiring a certain distance between retailers with tobacco licenses)[32, 33]. A combination of tobacco retailer licensing reduction policies could then be used to reduce TRA overall while centering equity. Still, in states without preemption, our study demonstrates that zoning policies may offer innovative and pro-equity approaches to reduce TRA in ways that other policies may not[20, 32, 34]. However, vigilance over the tobacco industry must be taken to prevent the preemption of zoning approaches.

Of importance, our study simulates the random removal of tobacco retailers in targeted zones to parallel real-world policies that implement a cap or limit on the number of retailers that are permitted to sell tobacco in a geographic area. Reductions in tobacco retailers through capping policies usually are the product of natural attrition of tobacco retailers due to things like store turnover, retailers choosing not paying tobacco retail licensing fees, or losing licenses due to violations (e.g., sales to minors). Like capping policies, the impact of zoning policies may initially only affect new retailers wanting to sell tobacco products rather than those already selling. However, amortization may be used, where, for example, retailers are permitted to sell tobacco for a certain period of time (e.g., 5 years) before the zoning policy is implemented to give time for retailers to transition and cease the sales of tobacco products[33, 39]. Amortization periods may also help with potential pushback on policies that reduce tobacco retailer sales. Finally, as zoning-based tobacco retailer reduction policies are innovative and have not been implemented or evaluated widely, future work understanding facilitators and barriers to the design, implementation, and evaluation is needed. Importantly, we emphasize that these policies should not aim to close businesses; rather, retailers would continue to operate but cease tobacco product sales. Incentive programs that work with businesses to provide financial or advertising incentives for ceasing the sales of tobacco products[65] may promote feasibility and adoption of these and other tobacco retailer reduction policies.

There are limitations to the current study. First, our study is based in OKC and Tulsa, two cities in a single state. Zoning and land use policies and regulations are highly localized, and our findings may not be generalizable to other places. Second, we prioritized zoning policies based on previous analyses indicating a larger number of zones and inequities in TRA[55], and we did not examine the impact of every potential zoning policy in these two cities. Third, as discussed above, our simulations randomly removed tobacco retailers to estimate the effects of zoning-based tobacco retailer reduction policies. This random removal may not represent how these types of policies would be designed and implemented in the real world, and they do not take into consideration how long it would take to observe the estimated policy effects in practice. Finally, we used a list of licensed tobacco retailers provided by the state, but we did not verify tobacco product sales, and there may be unlicensed tobacco retailers operating that were not captured in our sample.

## Conclusions

Our study provides promising evidence that zoning policies may reduce overall TRA and reduce racialized and socioeconomic inequities in TRA. Conducting equity assessments that examine neighborhood-level patterns between sociodemographic characteristics, tobacco retailer locations, and zoning and land use at the outset can inform the design of zoning policies to ensure inequities are not created or exacerbated. Still, state-level preemption of tobacco retailer reduction policies may stifle the impact of zoning and other tobacco retailer reduction policies, demonstrating the need to repeal preemption to allow the implementation of multiple tobacco control strategies. Overall, zoning policies may be an additional policy tool that can be used in combination with other tobacco retailer reduction policies to reduce tobacco-related morbidity and mortality, especially in preempted states and those neighborhoods experiencing an inequitable concentration of tobacco retailers.

## Supplementary Information

Below is the link to the electronic supplementary material.ESM 1(PDF 682 KB)

## Data Availability

Data used in this study may be requested or downloaded from the American Community Survey (U.S. Census Bureau), the Indian Nations Council of Governments (INCOG), the City of Oklahoma CIty City Open Data Portal, and the Oklahoma State Department of Health.
