# Supplementary material for: Simulating the Impact of Zoning Policies on Equitably Reducing Neighborhood Tobacco Retailer Availability
Source: J Urban Health. 2026 Jul 21;103(4):756–70. doi: 10.1007/s11524-026-01119-6 (PMC13524895; doi:10.1007/s11524-026-01119-6)
Supplement: Supplementary file 1 [file 11524_2026_1119_MOESM1_ESM.pdf]

Supplemental Figure 1. PUD (Planned Unit Development) tobacco control policy cap simulation: Estimated count of tobacco retailers remaining pre- vs. post-policy simulation by sociodemographic variable quintiles, Oklahoma City, Oklahoma

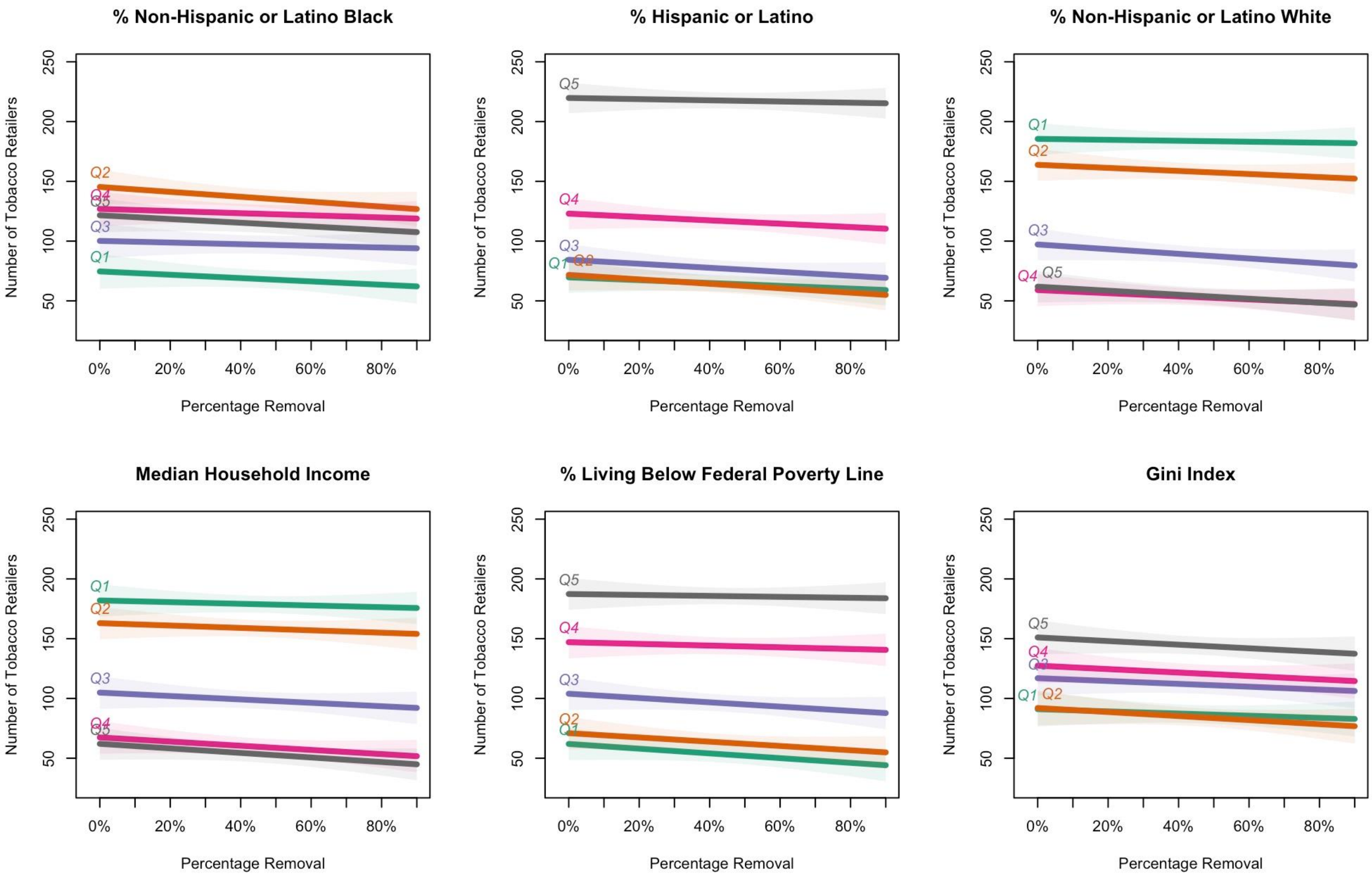

Note: Quintile (Q)1 represents the lowest value of the sociodemographic variable while Q5 represents the highest value of the sociodemographic variable.

Supplemental Figure 2. Light Industrial (I-1) tobacco control policy cap simulation: Estimated count of tobacco retailers remaining pre- vs. post-policy simulation by sociodemographic variable quintiles, Oklahoma City, Oklahoma

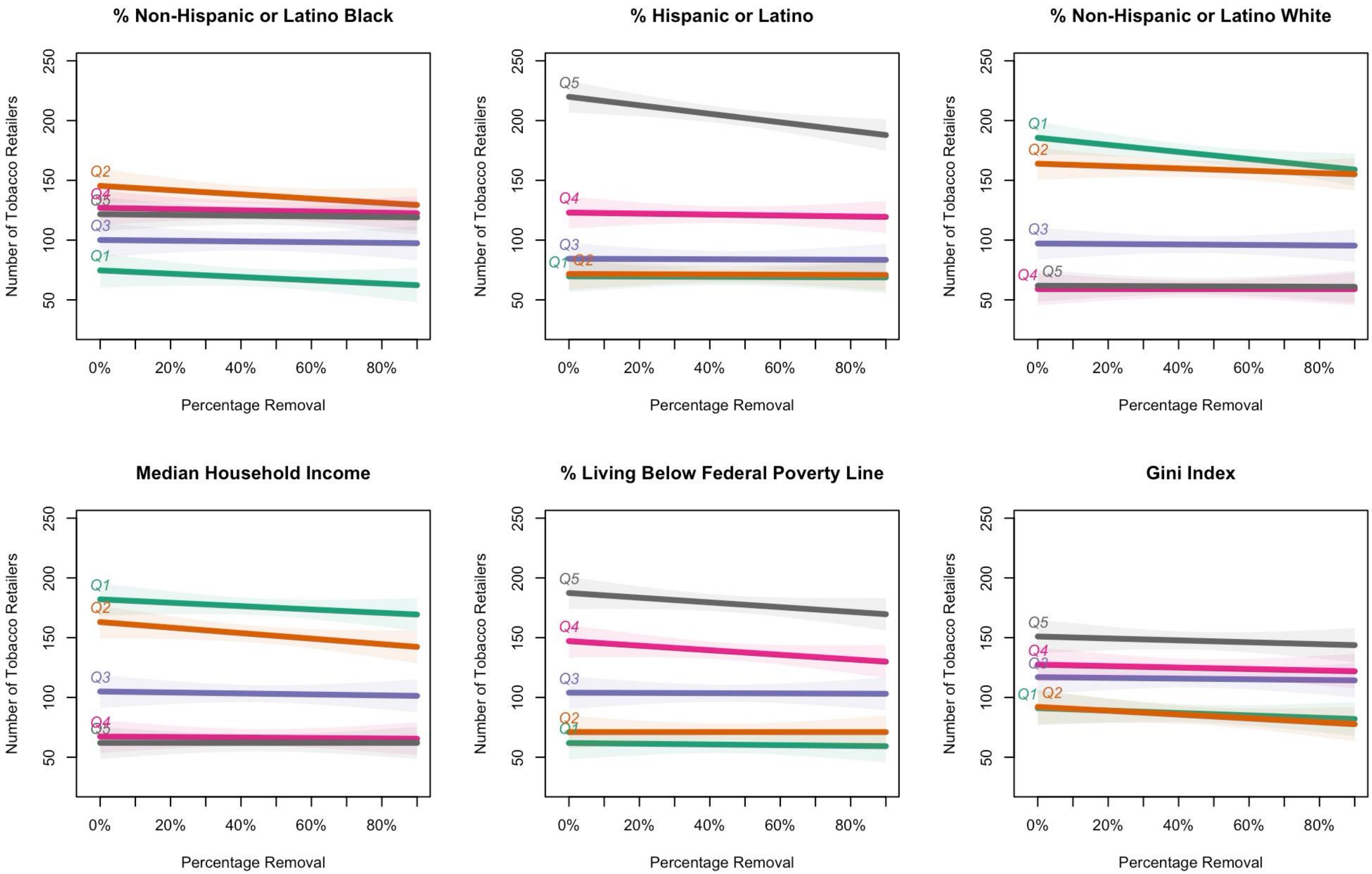

Note: Quintile (Q)1 represents the lowest value of the sociodemographic variable while Q5 represents the highest value of the sociodemographic variable.

Supplemental Figure 3. Moderate Industrial (I-2) tobacco control policy cap simulation: Estimated count of tobacco retailers remaining pre- vs. post-policy simulation by sociodemographic variable quintiles, Oklahoma City, Oklahoma

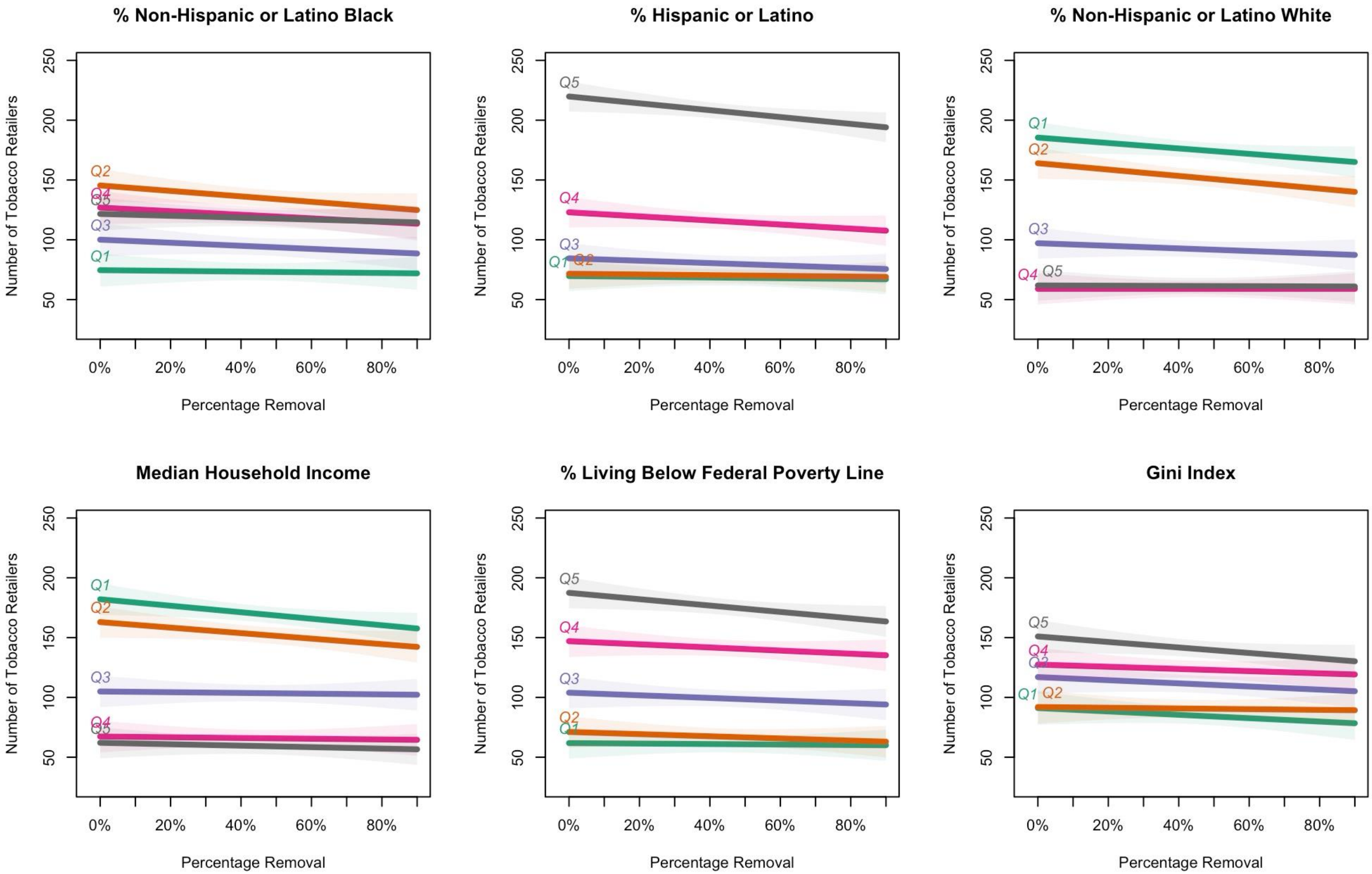

Note: Quintile (Q)1 represents the lowest value of the sociodemographic variable while Q5 represents the highest value of the sociodemographic variable.
